# Clinicopathological Characteristics and Prognostic Impact of KRAS Mutations in Non-Small Cell Lung Cancer

**DOI:** 10.3390/medicina62061011

**Published:** 2026-05-23

**Authors:** Tayyip İlker Aydın, Gökhan Öztürk, Aysun Fatma Akkuş, Ebru Taştekin, Sernaz Topaloğlu, Bülent Erdoğan, Ahmet Küçükarda, Muhammet Bekir Hacıoğlu

**Affiliations:** 1 Department of Medical Oncology, Trakya University Faculty of Medicine, 22030 Edirne, Türkiye; ilker6125@gmail.com (T.İ.A.); aysunfatmadogan@gmail.com (A.F.A.); sernaz.uzunoglu@gmail.com (S.T.); berdoga@hotmail.com (B.E.); ahmetkucukarda22@gmail.com (A.K.); mbekirhacioglu@yahoo.com (M.B.H.); 2 Department of Pathology, Trakya University Faculty of Medicine, 22030 Edirne, Türkiye; ebrutastekin@hotmail.com

**Keywords:** non-small cell lung cancer, KRAS mutation, programmed cell death ligand 1 protein (PD-L1), prognosis

## Abstract

*Background/Objectives*: KRAS mutations are among the most common oncogenic driver alterations in non-small cell lung cancer (NSCLC) and define a biologically heterogeneous disease. In the current era of molecular oncology, with targeted therapies increasingly incorporated into clinical practice, the prognostic relevance of individual KRAS mutation subtypes and their relationship with immune biomarkers such as programmed cell death ligand 1 (PD-L1) require further clarification. This study aimed to evaluate the prognostic impact of KRAS mutation subtypes and their association with PD-L1 expression in patients with NSCLC. *Methods*: In this retrospective analysis, 150 patients with KRAS-mutant NSCLC who underwent next-generation sequencing at Trakya University Faculty of Medicine between January 2015 and December 2023 were included. Clinicopathological features, KRAS mutation subtypes, PD-L1 expression, and survival outcomes were assessed. Overall survival (OS) and progression-free survival (PFS) were estimated using the Kaplan–Meier method, and prognostic factors were evaluated using Cox regression analyses. *Results*: KRAS G12C was the most frequent subtype (40.7%), followed by G12V (20.7%) and G12D (14.7%). OS differed significantly among KRAS mutation subtypes (log-rank *p* = 0.007), with median OS values of 18 months for G12D, 11 months for G12C, 11 months for other rare variants, 9 months for G12A and G12V, and 5 months for G13. PD-L1 positivity was significantly higher in KRAS G12C tumors compared with non-G12C subtypes and remained independently associated with improved OS in multivariate Cox regression analysis (HR = 0.622; 95% CI, 0.426–0.907; *p* = 0.014). In multivariate analysis, age, ECOG performance status, disease stage, and PD-L1 positivity were independent prognostic factors, whereas KRAS mutation subtype did not retain independent prognostic significance. *Conclusions*: These findings suggest that KRAS-mutant NSCLC represents a clinically and molecularly heterogeneous subgroup and that integrating KRAS mutation subtypes with immune biomarkers may support more refined prognostic stratification.

## 1. Introduction

Lung cancer remains the leading cause of cancer-related mortality worldwide and accounts for the largest proportion of cancer-related deaths each year. Non-small cell lung cancer (NSCLC) constitutes approximately 85% of all lung cancer cases and represents the most common histological subtype [1]. Advances in molecular biology over the past decade have improved our understanding of the heterogeneous nature of NSCLC and have facilitated the identification of key driver mutations involved in its pathogenesis [2]. This molecular subclassification has become critically important not only for elucidating disease biology but also for prognostic and predictive assessment.

Among the identified driver mutations, Kirsten rat sarcoma viral oncogene homolog (KRAS) is one of the most frequently observed oncogenic alterations in NSCLC and is detected in approximately 20–25% of lung adenocarcinomas [3]. KRAS mutations are predominantly clustered around codons 12 and 13 and constitute a distinct clinical subgroup because of their high prevalence and marked biological and clinical heterogeneity. Although the distribution of KRAS subtypes varies across populations, G12C is the most commonly reported variant and is typically associated with smoking exposure [3]. In addition, G12D and G12V mutations are also among the frequently reported subtypes. Increasing evidence suggests that these molecular variants differ in terms of tumor biology, interactions with the tumor microenvironment, and clinical outcomes [4]. Although several studies have suggested that individual KRAS subtypes, including G12D and G12V, may be associated with distinct survival outcomes, the prognostic value of these variants remains uncertain because current evidence is inconsistent and largely derived from retrospective cohorts [5].

The heterogeneous nature of KRAS-mutant NSCLC is not limited to the primary KRAS alteration but is further shaped by co-occurring genetic alterations such as TP53, STK11/LKB1, and KEAP1. This complexity complicates the prediction of treatment responses and increases the need for more refined biomarkers [6]. In recent years, the clinical introduction of selective inhibitors targeting KRAS G12C, particularly sotorasib and adagrasib, has expanded targeted treatment options for patients with KRAS-mutant NSCLC [7]. This therapeutic progress underscores the importance of evaluating KRAS subtypes not only from a biological perspective but also in terms of their clinical and therapeutic implications. Taken together, these data indicate that KRAS-mutant NSCLC does not represent a uniform disease entity but rather a spectrum characterized by marked molecular and clinical heterogeneity. Within this context, the present study aimed to comprehensively evaluate the associations between KRAS mutation subtypes, co-occurring molecular alterations, and clinical outcomes in patients with KRAS-mutant NSCLC.

## 2. Methods

This retrospective study included 150 patients with KRAS-mutant non-small cell lung cancer (NSCLC) who were treated and followed at Trakya University Faculty of Medicine between January 2015 and December 2023 and who underwent next-generation sequencing (NGS) analysis. The study was conducted in accordance with the Declaration of Helsinki and received approval from the Ethics Committee of Trakya University Faculty of Medicine (Approval No: 2025/546; approval date: 15 December 2025).

### 2.1. Patient Selection and Data Collection

Patients with histologically confirmed NSCLC who were found to harbor a KRAS mutation by next-generation sequencing (NGS) performed on formalin-fixed, paraffin-embedded (FFPE) tumor tissue were screened for eligibility. Patients were included if complete baseline clinicopathological information and survival data were available. Patients were excluded if they had missing or inaccessible survival data; concurrent actionable driver alterations other than KRAS, including EGFR, ALK, ROS1, RET, or MET exon 14 skipping alterations; or a history of a second primary malignancy within the previous five years.

Demographic, clinical, and pathological data—including age, sex, smoking history, ECOG performance status, disease stage, metastatic sites (brain, bone, pleura/contralateral lung, visceral organs, and distant lymph nodes), and systemic treatment information—were obtained from institutional electronic medical records.

### 2.2. PD-L1 Immunohistochemistry

PD-L1 expression was evaluated by immunohistochemistry using the Ventana SP263 antibody on the Ventana BenchMark ULTRA automated staining platform (Ventana Medical Systems, Inc., Tucson, AZ, USA). The Tumor Proportion Score (TPS) was defined as the percentage of viable tumor cells showing partial or complete membranous staining.

PD-L1 expression was classified as <1% (negative), 1–49% (low positive), and ≥50% (high positive), and overall PD-L1 positivity was defined as TPS ≥ 1%.

### 2.3. Molecular Analysis

Molecular profiling was performed at the Molecular Pathology Laboratory of Trakya University Health Research and Application Center using the QIAseq Targeted DNA Custom Lung Panel (QIAGEN, Germantown, MD, USA) on the Illumina NextSeq 550 platform.

DNA was isolated from formalin-fixed, paraffin-embedded (FFPE) tumor sections containing ≥50% tumor cells and quantified using a Qubit 2.0 Fluorometer (Thermo Fisher Scientific, Waltham, MA, USA). Library preparation and target enrichment were conducted in accordance with the manufacturer’s instruction.

Sequencing data were analyzed using QIAGEN Clinical Insight (QCI) Analyze Universal and QCI Interpret software (QIAGEN, Hilden, Germany; QCI Interpret version 7.1.20210316), which incorporate curated databases including ClinVar, COSMIC, TCGA, and gnomAD. The targeted lung panel covered 56 genes relevant to lung cancer, including KRAS, TP53, STK11, KEAP1, PIK3CA, RICTOR, BRAF, NRAS, and EGFR, and also included a 26-gene microsatellite instability (MSI) panel.

### 2.4. Statistical Analysis

All statistical analyses were performed using IBM SPSS Statistics for Windows, version 22 (IBM Corp., Armonk, NY, USA). Continuous variables were summarized as mean ± standard deviation, and categorical variables as frequencies and percentages. The normality of continuous variables was assessed using visual methods and descriptive statistics.

For comparisons between two groups, Student’s *t*-test was used for normally distributed continuous variables. One-way analysis of variance (ANOVA) was applied for comparisons involving more than two groups. Categorical variables were compared using the Pearson chi-square test or Fisher’s exact test, as appropriate.

Overall survival (OS) was defined as the time from the date of diagnosis to death from any cause or last follow-up, whereas progression-free survival (PFS) was defined as the time from the initiation of first-line treatment to documented disease progression or death. Survival curves were estimated using the Kaplan–Meier method, and differences between groups were compared using the log-rank test. Prognostic factors for OS and PFS were evaluated using univariate and multivariate Cox proportional hazards regression analyses. The proportional hazards assumption was assessed visually using log-minus-log survival plots for categorical variables included in the Cox regression models. Formal interaction terms were not included in the Cox models because of the limited subgroup sizes in several KRAS mutation categories, which could have increased the risk of model overfitting and unstable estimates. No formal correction for multiple testing was applied in subgroup analyses; therefore, subgroup findings were interpreted as exploratory.

All statistical tests were two-sided, and a *p* value < 0.05 was considered statistically significant.

## 3. Results

### 3.1. Baseline Characteristics

A total of 150 patients were included in the study. The cohort consisted of 84% men (n = 126) and 16% women (n = 24). With respect to smoking history, 56% of patients were current smokers, 30% were former smokers, and 14% had never smoked. The median age at diagnosis was 62 years (range, 38–83). Regarding performance status, ECOG scores were 0 in 40.7% of patients, 1 in 52.7%, 2 in 6.0%, and 3 in 0.7%. The distribution of disease stage at diagnosis was as follows: stage I, 4.0%; stage II, 3.3%; stage III, 26.0%; and stage IV, 66.7%. Adenocarcinoma histology was present in 96% of patients.

Molecular profiling showed that KRAS G12C was the most frequent subtype, accounting for 40.7% of cases, followed by G12V (20.7%), G12D (14.7%), G12A (7.3%), G13 (7.3%), and other rare point mutations (9.3%). Co-occurring mutations were detected in PIK3CA, TP53, STK11, and KEAP1, with frequencies of 12%, 10%, 5.3%, and 2.7%, respectively. In terms of PD-L1 expression, 46.7% of tumors had a TPS < 1%, 32.0% had a TPS of 1–49%, and 14.7% had a TPS ≥ 50%. Accordingly, the overall PD-L1 positivity rate (TPS ≥ 1%) was calculated as 46.7%. PD-L1 status was unknown in 10 patients (6.7%) because PD-L1 testing had not been performed (Table 1).

### 3.2. Analysis of KRAS Mutation Subtypes

No statistically significant differences were observed among KRAS mutation subtypes with respect to sex (*p* = 0.420), age (*p* = 0.140), ECOG performance status (*p* = 0.667), smoking history (*p* = 0.420), PIK3CA mutation status (*p* = 0.343), or KEAP1 mutation status (*p* = 0.503). Similarly, no significant differences were found in the presence of distant lymph node (*p* = 0.855), brain (*p* = 0.787), bone (*p* = 0.782), pleural or contralateral lung (*p* = 0.782), or visceral metastases (*p* = 0.847).

In patients harboring KRAS G12C mutations, metastatic involvement rates were 47.5% for the brain, 50.8% for bone, 49.2% for pleural or contralateral lung, 52.5% for visceral organs, and 41.0% for distant lymph nodes. In contrast, the corresponding rates in the non-G12C group were 37.1%, 46.1%, 55.1%, 53.9%, and 46.1%, respectively. Although brain and bone metastases appeared numerically more frequent in the KRAS G12C subgroup, these differences did not reach statistical significance (all *p* > 0.05). Similarly, the distribution of disease stage at diagnosis did not differ significantly between the two groups (*p* = 0.293). In the KRAS G12C cohort, 68.9% of patients presented with stage IV disease, 26.2% with stage III, and 4.9% with stage I disease, whereas in the non-G12C group, the proportions of stage IV, III, II, and I disease were 65.2%, 25.8%, 5.6%, and 3.4%, respectively.

Among patients receiving first-line systemic therapy with chemotherapy alone or chemo-immunotherapy, first-line systemic treatment distribution did not differ significantly across KRAS mutation subtypes (Fisher’s exact test, *p* = 0.690). Similarly, no significant difference in first-line systemic treatment distribution was observed between the KRAS G12C and non-G12C subgroups (chemo-immunotherapy: 7.1% vs. 5.1%, respectively; Fisher’s exact test, *p* = 0.691).

**Figure 1:**
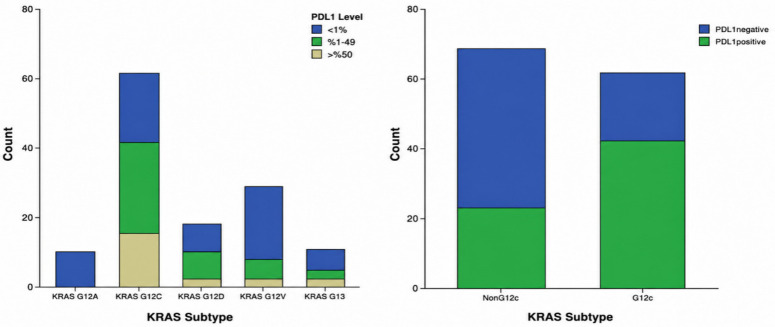
Distribution of PD-L1 expression levels according to KRAS mutation subtypes (**left**) and comparison of PD-L1 positivity between KRAS G12C and KRAS non-G12C groups (**right**).

A significant association was observed between KRAS mutation subtype and PD-L1 expression (*p* < 0.001). PD-L1 positivity was markedly higher in the KRAS G12C group (68.9%) compared with other subtypes (35.4%). This association remained significant when KRAS status was dichotomized as G12C versus non-G12C (*p* < 0.001) (Figure 1).

**Figure 2:**
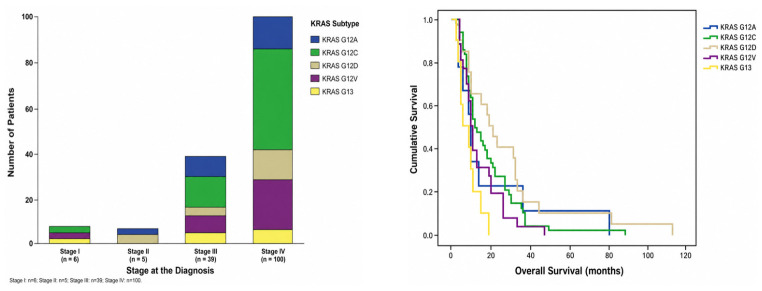
Distribution of clinical stage at diagnosis (**left**) and Kaplan–Meier overall survival curves (**right**) according to KRAS mutation subtypes.

According to Kaplan–Meier survival analysis, the median overall survival (OS) for the entire cohort was 10 months (95% CI, 8.1–11.9). Median OS differed across KRAS mu tation subtypes: 18 months for G12D, 11 months for G12C, 11 months for other rare vari ants, 9 months for G12A and G12V, and 5 months for G13. This difference was statistically significant in the log-rank test (*p* = 0.007) (Figure 2). However, patients with KRAS G12C mutations had only a numerically longer OS than those with non-G12C variants, and this difference was not statistically significant (median OS: 11 vs. 9 months; log-rank *p* = 0.530).

#### 3.2.1. PD-L1 Expression Analysis

No significant associations were observed between PD-L1 expression and sex (*p* = 0.813), age (*p* = 0.928), smoking status (*p* = 0.343), disease stage at diagnosis (*p* = 0.871), or the presence of brain (*p* = 0.731), bone (*p* = 0.612), or visceral metastases (*p* = 0.236). Although PD-L1 positivity was numerically higher in patients with pleural or contralateral lung metastases, this difference did not reach statistical significance (*p* = 0.062). When analyzed according to KRAS mutation status, PD-L1 positivity was significantly higher in the KRAS G12C cohort than in the non-G12C cohort (68.9% vs. 35.4%, *p* < 0.001).

Among patients with available PD-L1 status who received first-line systemic therapy with chemotherapy alone or chemo-immunotherapy, first-line treatment distribution did not differ significantly according to PD-L1 status. Chemo-immunotherapy was administered to 8.5% of PD-L1–positive patients and 2.1% of PD-L1–negative patients (Fisher’s exact test, *p* = 0.204). Similarly, among patients receiving second-line systemic therapy, the distribution of chemotherapy and immunotherapy did not differ significantly according to PD-L1 status. Second-line immunotherapy was administered to 20.8% of PD-L1–positive patients and 34.8% of PD-L1–negative patients (Fisher’s exact test, *p* = 0.341).

**Figure 3:**
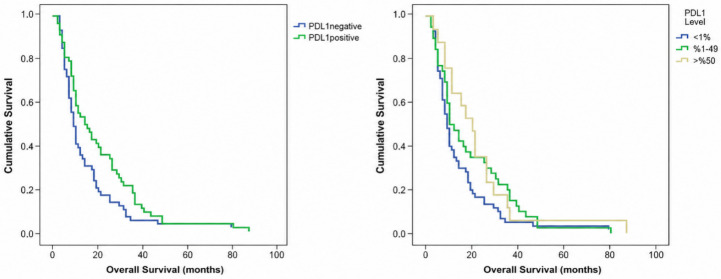
Kaplan–Meier overall survival curves according to PD-L1 expression status (**left**) and PD-L1 expression levels (**right**) in the study cohort.

In survival analyses, the median overall survival (OS) was 14 months in PD-L1–positive patients and 9 months in PD-L1–negative patients (log-rank *p* = 0.029). When PD-L1 expression was categorized as <1%, 1–49%, and ≥50%, the difference in OS did not reach statistical significance (*p* = 0.081); however, median OS increased stepwise across PD-L1 expression categories, from 9 months in the <1% group to 10 months in the 1–49% group and 20 months in the ≥50% group (Figure 3).

#### 3.2.2. Cox Regression Analysis

In univariate Cox regression analysis, age, ECOG performance status, disease stage at diagnosis, and PD-L1 positivity were significantly associated with overall survival (*p* < 0.05) (Table 2). Other clinical, molecular, and metastatic variables, including KRAS mutation subtypes, did not show a statistically significant association with overall survival. Thus, although KRAS mutation subtypes showed significant OS differences in Kaplan–Meier analysis, this association was not confirmed in Cox regression analysis, and KRAS subtype was not retained as an independent prognostic factor in the multivariate model.

When these variables were included in the multivariate Cox regression model, age (HR = 1.032; 95% CI, 1.005–1.060; *p* = 0.018), ECOG performance status (HR = 1.697; 95% CI, 1.223–2.354; *p* = 0.002), disease stage at diagnosis (HR = 2.429; 95% CI, 1.692–3.486; *p* < 0.001), and PD-L1 positivity (HR = 0.622; 95% CI, 0.426–0.907; *p* = 0.014) remained independently associated with OS. Increasing age, poorer performance status, and more advanced disease stage were independently associated with worse survival, whereas PD-L1 positivity was associated with better survival outcomes. Visual inspection of the log-minus-log survival plots for categorical variables included in the Cox regression models did not indicate a substantial violation of the proportional hazards assumption.

### 3.3. Clinical and Survival Characteristics of Patients with Metastatic Disease

Among the 150 patients included in the study, 138 (92.0%) presented with metastatic disease, of whom 100 (66.7%) had de novo metastatic disease and 38 (25.3%) had recurrent metastatic disease. Among patients with metastatic disease, 93% received platinum-based chemotherapy as first-line treatment, whereas 7% were treated with a chemo-immunotherapy combination. Second-line therapy was administered to 52.7% of patients, including chemotherapy in 40.0% and immunotherapy in 12.7%. The rates of third- and fourth-line treatment were 22.8% and 10.1%, respectively.

The most common sites of metastasis were visceral organs (53.3%), pleura or contralateral lung (52.7%), bone (48.0%), distant lymph nodes (44.0%), and brain (41.3%).

**Figure 4:**
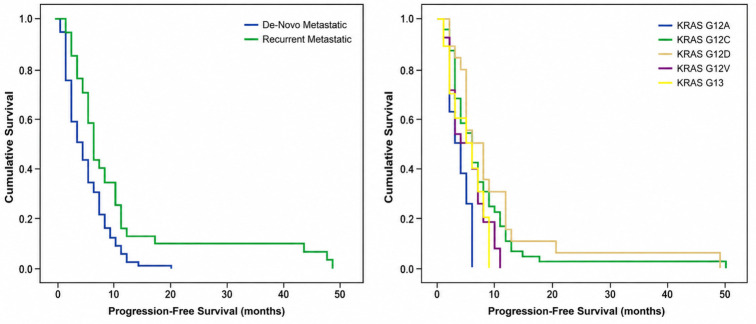
Kaplan–Meier curves showing progression-free survival (PFS) according to metastatic pattern (**left**) and KRAS mutation subtypes (**right**).

In Kaplan–Meier analysis, the median progression-free survival (PFS) for the entire metastatic cohort was 5.0 months (95% CI, 3.9–6.0). PFS was significantly longer in patients with recurrent metastatic disease than in those with de novo metastatic disease (7.0 vs. 4.0 months, *p* = 0.002) (Figure 4). According to PD-L1 expression status, median PFS was 7.0 months in PD-L1–positive tumors and 4.0 months in PD-L1–negative tumors (*p* = 0.018). When analyzed by KRAS mutation subtype, a significant difference in PFS was observed (*p* = 0.032), with the longest median PFS observed in KRAS G12D (7.0 months) and the shortest observed in KRAS G12A (3.0 months).

In univariate Cox regression analysis, ECOG performance status (*p* = 0.002), PD-L1 positivity (*p* = 0.032), and metastatic status (*p* = 0.005) were significantly associated with PFS, whereas age (*p* = 0.279), sex (*p* = 0.988), KRAS mutation subtype (*p* = 0.680), and TP53 mutation status (*p* = 0.821) were not. In the multivariate model, ECOG performance status (HR = 1.40; 95% CI, 1.01–1.95; *p* = 0.046) and metastatic status (HR = 0.62; 95% CI, 0.40–0.97; *p* = 0.037) remained independent prognostic factors for PFS, while PD-L1 positivity (*p* = 0.085) and brain metastasis (*p* = 0.165) showed trends that did not reach statistical significance.

## 4. Discussion

This study reflects real-world experience from a tertiary referral center with access to next-generation sequencing–based molecular profiling, providing insight into the clinicopathological characteristics and outcomes of KRAS-mutant NSCLC in routine clinical practice, where access to comprehensive genomic testing may vary across clinical settings. Significant differences in overall survival (OS) were observed among KRAS mutation subtypes (log-rank *p* = 0.007), with median OS values of 18 months for G12D, 11 months for G12C, 11 months for other rare variants, 9 months for G12A and G12V, and 5 months for G13. In the metastatic subgroup, PFS also differed significantly according to KRAS subtype (*p* = 0.032), with median PFS values ranging from 3.0 months in the G12A subgroup to 7.0 months in the G12D subgroup. Notably, a significant association was identified between KRAS mutation subtypes and PD-L1 expression, with PD-L1 positivity being significantly higher in the KRAS G12C group (*p* < 0.001). In survival analyses, PD-L1–positive patients exhibited longer overall survival (14 months vs. 9 months, *p* = 0.029), and PD-L1 positivity remained independently associated with improved OS in multivariate Cox regression analysis (HR = 0.622; 95% CI, 0.426–0.907; *p* = 0.014).

KRAS functions as a central signaling node in intracellular signal transduction and is dynamically regulated between its GDP-bound inactive and GTP-bound active forms through upstream signals initiated by receptor tyrosine kinases of the EGFR family [8]. Oncogenic KRAS mutations disrupt this physiological regulation, leading to constitutive activation of the MAPK and PI3K–AKT signaling pathways. This aberrant signaling promotes increased cellular proliferation, survival, and therapeutic resistance [8], establishing KRAS as a key molecular driver in tumor development and progression.

Beyond this core signaling mechanism, accumulating evidence indicates that KRAS-mutant tumors do not represent a single molecular entity but rather comprise multiple distinct mutation subtypes with potentially different biological and clinical behaviors [9]. These subtype-specific alterations may contribute to intratumoral heterogeneity and differential evolutionary dynamics within KRAS-mutant tumors, highlighting the biological complexity of this molecular subgroup and providing a rationale for investigating potential clinical differences among individual KRAS mutation variants [10]. Although the biological heterogeneity of KRAS-mutant NSCLC is widely recognized, the prognostic implications of individual KRAS mutation subtypes remain inconsistent across studies. Several reports have suggested subtype-specific differences in clinical outcomes, but the direction and magnitude of these associations have varied considerably according to study population and disease setting. In a large metastatic cohort, the poorest overall survival was observed in the KRAS G13 subgroup, whereas G12C was the most prevalent variant [5]. By contrast, Cai et al. reported inferior overall and progression-free survival in patients harboring KRAS G12D mutations within a uniformly metastatic, chemotherapy-treated population, while Gu et al. found that G12C and G12V were associated with higher PD-L1 expression, greater immunotherapy benefit, and more favorable PFS and OS than G12D, suggesting that specific KRAS variants may differ not only in prognosis but also in immune contexture and treatment responsiveness [11,12]. Similarly, Liu et al. emphasized that the adverse survival effect observed in KRAS-mutant disease was largely driven by the G12V and G12D subgroups [13]. However, not all studies have demonstrated clear prognostic separation between subtypes; in a multicenter analysis, no significant differences in OS or PFS were observed between G12C and non-G12C tumors [14]. Climent et al. further highlighted that subtype-specific stage distribution may substantially influence survival interpretation, reporting a higher proportion of early-stage disease in the G12D subgroup and more frequent metastatic presentation in G12C and G12V tumors [15]. In line with this heterogeneous literature, our analysis also demonstrated significant variation in median OS across KRAS mutation subtypes in the Kaplan–Meier analysis. Notably, the unfavorable outcome observed in patients with G13-mutant tumors was broadly aligned with previous reports, whereas patients harboring G12D alterations in our cohort showed relatively more favorable survival, in contrast to some metastatic-only cohorts in which G12D has been associated with inferior outcomes [5,11]. This discrepancy may partly reflect differences in stage distribution, disease setting, and clinical context across studies. Nevertheless, although OS differed significantly among KRAS mutation subtypes in Kaplan–Meier analysis, KRAS subtype did not retain statistical significance in the multivariate Cox model. This finding should not be interpreted as definitive evidence against a potential biological or prognostic role of KRAS subtypes, particularly given the limited sample sizes within several mutation categories and the resulting constraints in statistical power. Although measured baseline clinical variables, including ECOG performance status, disease stage, metastatic pattern, and major co-mutations, did not differ significantly across KRAS subtypes, the relatively small number of patients within subtype-specific clinical strata limits our ability to fully exclude residual confounding. In addition, differences in stage distribution, baseline performance status, treatment exposure, access to subsequent lines of therapy, and the limited use of contemporary chemo-immunotherapy or KRAS G12C–targeted agents may have influenced OS estimates in this real-world cohort. Therefore, the Kaplan–Meier differences observed in our study should be regarded as hypothesis-generating and descriptive, rather than as definitive evidence that individual KRAS subtypes independently determine prognosis.

Among KRAS mutation subtypes, the G12C variant has attracted particular clinical attention because of its relatively high prevalence and emerging therapeutic implications. The development of selective covalent inhibitors targeting KRAS G12C has fundamentally altered the therapeutic landscape of KRAS-mutant NSCLC, establishing this variant as one of the few directly druggable oncogenic drivers in lung cancer. In the phase 2 CodeBreaK-100 study, the KRAS G12C inhibitor sotorasib demonstrated an objective response rate of approximately 37% and a median progression-free survival of 6.8 months in previously treated patients with KRAS G12C–mutant NSCLC, representing a meaningful improvement over historical second-line chemotherapy outcomes [7]. Similar clinical activity has also been observed with other KRAS G12C inhibitors, further reinforcing the clinical relevance of this mutation subtype [16]. Beyond its therapeutic implications, several studies suggest that KRAS G12C tumors may exhibit distinct molecular characteristics compared with other KRAS variants. For example, Gu et al. reported that KRAS G12C mutations were more frequently associated with PIK3CA co-mutations and were more commonly observed in smokers, and were also associated with higher tumor mutational burden compared with several other KRAS variants [12]. These molecular differences may contribute to the biological diversity observed across KRAS-mutant NSCLC. However, clinical outcomes do not always differ substantially between G12C and other KRAS variants. In a multicenter study including 101 patients with metastatic KRAS-mutant NSCLC, Aytaç et al. reported that although brain metastases were numerically less frequent in patients with KRAS G12C mutations compared with non-G12C variants (33.3% vs. 43.8%), no statistically significant differences were observed between the two groups in terms of response rates, progression-free survival, or overall survival [14]. In our cohort, KRAS G12C represented the most common subtype, accounting for 40.7% of all KRAS mutations. No statistically significant differences were observed between G12C and non-G12C tumors with respect to baseline clinical characteristics, metastatic distribution, or stage at diagnosis. In survival analyses, patients with KRAS G12C mutations demonstrated a numerically longer overall survival compared with those with non-G12C variants (median OS: 11 vs. 9 months), although this difference did not reach statistical significance (log-rank *p* = 0.530). Importantly, no patient in our cohort received KRAS G12C inhibitor therapy; therefore, survival comparisons between G12C and non-G12C tumors should be interpreted without the confounding influence of contemporary KRAS G12C–targeted therapies. Nevertheless, the rapidly evolving therapeutic landscape of KRAS G12C–targeted therapies underscores the importance of evaluating this mutation separately from other KRAS variants. Taken together, these observations support the notion that KRAS G12C–mutant NSCLC may constitute a distinct molecular subset in the era of emerging KRAS-targeted therapies.

Beyond the subtype-specific therapeutic relevance of KRAS G12C, its interplay with the tumor immune microenvironment is also of considerable interest, particularly with respect to PD-L1 expression. In the current era of immunotherapy and molecularly stratified treatment, PD-L1 remains one of the most clinically relevant biomarkers in NSCLC, not only for treatment selection but also, in selected contexts, for prognostic stratification. However, the prognostic significance of PD-L1 in NSCLC has been inconsistent across studies. In a retrospective study of 97 patients, Zhao et al. reported that PD-L1 positivity was associated with more aggressive pathological features and poorer prognosis in advanced-stage NSCLC, with PD-L1 positivity in advanced disease associated with inferior outcome (HR: 4.13; 95% CI: 1.06–16.12) [17]. Similarly, in a 5-year follow-up study, Huang et al. found that PD-L1–positive NSCLC was associated with shorter survival than PD-L1–negative disease and remained an independent adverse prognostic factor [18]. By contrast, in advanced KRAS-mutant NSCLC treated with first-line immunochemotherapy, Jin et al. showed a stepwise improvement in progression-free survival according to PD-L1 level in primary lung lesions, from 7.27 months in tumors with PD-L1 < 1% to 8.30 months in those with PD-L1 1–49% and 15.00 months in those with PD-L1 ≥ 50% (*p* < 0.001), highlighting the context-dependent prognostic and predictive relevance of PD-L1 in this molecular subgroup [19]. Within KRAS-mutant NSCLC, accumulating evidence further suggests that PD-L1 expression may vary across KRAS subtypes. Gu et al. reported that G12C and G12V tumors were associated with increased PD-L1 expression and more favorable immunotherapy outcomes than G12D tumors, whereas Huang et al. observed significantly higher PD-L1 expression in KRAS G12C tumors than in non-G12C variants (*p* = 0.01), with better overall and progression-free survival among patients with high PD-L1 expression regardless of KRAS mutation type [12,18]. Consistent with this broader concept of immune heterogeneity, Yang et al. demonstrated subtype-specific differences in PD-L1 and TMB profiles across KRAS-mutant NSCLC, with decreased PD-L1 expression in KRAS Q61X tumors and increased PD-L1 expression in KRAS G13X tumors [20]. Nevertheless, not all studies have reported the same directional association; Aytaç et al. found no statistically significant difference in PD-L1 expression between G12C and non-G12C tumors, while Climent et al. observed lower PD-L1 positivity in the G12C subgroup and suggested that the apparent inverse relationship between PD-L1 expression and survival may have been influenced by subtype composition, particularly the overrepresentation of the biologically more aggressive G12V subtype [14,15]. In our cohort, PD-L1 positivity was significantly more frequent in KRAS G12C tumors than in non-G12C variants (68.9% vs. 35.4%, *p* < 0.001). Moreover, PD-L1 positivity was associated with longer OS in Kaplan–Meier analysis (14 vs. 9 months, *p* = 0.029) and remained independently associated with improved OS in multivariate Cox regression analysis (HR=0.622; 95% CI, 0.426–0.907; *p* = 0.014). In the metastatic cohort, PD-L1 positivity was also associated with longer PFS in Kaplan–Meier analysis (7 vs. 4 months, *p* = 0.018), although this association did not retain statistical significance in the multivariate model. When PD-L1 expression was stratified by level, a stepwise increase in median overall survival was observed from 9 months in the PD-L1–negative group to 10 months in the 1–49% group and 20 months in the ≥50% group, although this trend did not reach statistical significance (*p* = 0.081). Taken together, these findings suggest that PD-L1 expression represents an important layer of immune-related heterogeneity within KRAS-mutant NSCLC. However, the favorable survival association observed in our cohort should be interpreted cautiously, as PD-L1 positivity may not necessarily reflect a direct prognostic effect but may instead capture broader biological features linked to KRAS subtype distribution, co-mutational context, and tumor immune phenotype, particularly in KRAS G12C–mutant tumors.

Treatment exposure should also be considered when interpreting the relationship between KRAS mutation subtypes, PD-L1 expression, and survival outcomes in this real-world cohort. However, first-line systemic treatment was predominantly chemotherapy-based, with only a small proportion of patients receiving chemo-immunotherapy. Importantly, first-line systemic treatment distribution did not differ significantly across KRAS mutation subtypes or between KRAS G12C and non-G12C subgroups. In addition, first-line treatment distribution did not differ significantly according to PD-L1 status, and among patients receiving second-line systemic therapy, the distribution of chemotherapy and immunotherapy was also similar between PD-L1–positive and PD-L1–negative patients. These findings suggest that the observed association between PD-L1 positivity and improved OS is unlikely to be explained solely by preferential immunotherapy use in PD-L1–positive patients. Nevertheless, given the retrospective design, limited number of patients receiving chemo-immunotherapy, and potential influence of treatment eligibility, access to subsequent lines of therapy, and calendar-period effects, residual treatment-related confounding cannot be completely excluded.

Building upon the subtype-specific differences observed for KRAS G12C and the immune-related patterns associated with PD-L1 expression, it is also important to consider how clinical presentation—particularly disease stage and metastatic distribution—interacts with the biological heterogeneity of KRAS-mutant NSCLC. In this real-world cohort, the majority of patients presented with advanced disease, with approximately two-thirds diagnosed at stage IV, reflecting the well-recognized tendency of KRAS-mutant lung cancer to present with metastatic disease. Similar patterns have been reported in previous studies; for instance, Aytaç et al. reported that nearly 79% of KRAS-mutant patients presented with stage IV disease at diagnosis, while Climent et al. observed metastatic disease in approximately 65% of cases across KRAS subtypes [14,15]. In our analysis, although the distribution of metastatic sites did not differ significantly between KRAS G12C and non-G12C tumors, brain and bone metastases were numerically more frequent in the G12C subgroup, suggesting potential subtype-related differences in metastatic tropism that may not reach statistical significance due to limited subgroup sizes. Similarly, Climent et al. reported that metastatic disease was more frequently observed among G12C and G12V tumors compared with G12D mutations, whereas the latter subgroup tended to present with earlier-stage disease [15]. Together, these observations support the concept that clinical presentation patterns—including stage distribution and metastatic dissemination—may vary across KRAS mutation subtypes and contribute to the heterogeneity of clinical outcomes. Beyond these clinical differences, an additional layer of heterogeneity arises from the molecular context in which KRAS mutations occur. Co-occurring genomic alterations such as TP53, STK11, and KEAP1 are known to substantially influence tumor biology, immune microenvironment characteristics, and therapeutic responsiveness in KRAS-mutant lung cancer. In the large genomic analysis by Arbour et al., TP53, STK11, and KEAP1 were identified as the most frequent co-mutations accompanying KRAS alterations [21]. Notably, KEAP1 co-mutations were associated with inferior responses to chemotherapy and significantly shorter overall survival, highlighting the biological relevance of these molecular interactions. Similarly, Cai et al. reported that patients harboring concurrent TP53 and STK11 mutations exhibited the poorest progression-free and overall survival among KRAS-mutant tumors [11]. Additional studies have further demonstrated subtype-specific associations between KRAS mutations and co-mutation patterns; for example, Gu et al. found that G12C mutations were more frequently associated with PIK3CA alterations, whereas G12D tumors more commonly co-occurred with STK11 mutations [12]. Emerging transcriptomic analyses have also highlighted the molecular complexity of KRAS-mutant lung cancer. In a recent machine-learning-based study, Yang et al. identified three distinct molecular subtypes of KRAS G12C-mutant lung adenocarcinoma characterized by different immune microenvironment profiles, co-mutation patterns, and therapeutic vulnerabilities, further underscoring that KRAS-mutant tumors represent a biologically diverse group rather than a single molecular entity [22]. In line with these observations, recent evidence has positioned STK11 and KEAP1 alterations as key determinants of resistance to PD-(L)1 blockade, reflecting an immunologically “cold” tumor microenvironment and impaired antitumor immune responses. However, although co-mutation patterns may biologically contribute to treatment sensitivity and clinical outcomes in KRAS-mutant NSCLC, this could not be formally demonstrated in our cohort because of the limited number of patients harboring individual co-mutations. Accordingly, co-mutations such as TP53, STK11, and KEAP1 were descriptively reported but were not incorporated into the multivariate survival models, as their low individual frequencies, particularly for STK11 and KEAP1, would have increased the risk of overfitting and unstable effect estimates.

Within this broader biological framework, the findings from our cohort further support the concept of substantial clinical and molecular heterogeneity among KRAS-mutant tumors. Although no statistically significant associations were observed between KRAS mutation subtypes and baseline clinical characteristics or patterns of metastatic spread, the numerical differences observed in metastatic distribution and stage at diagnosis suggest that subtype-specific clinical behaviors may still exist, although their statistical demonstration may be limited by subgroup sizes. In parallel, the co-mutation spectrum identified in our cohort—most commonly involving PIK3CA, TP53, STK11, and KEAP1—was consistent with previously reported genomic patterns in KRAS-mutant NSCLC.

This study has several limitations that should be considered when interpreting the findings. First, the retrospective, single-center design may limit the generalizability of the results. Although our cohort represents a relatively large, well-characterized real-world series of KRAS-mutant NSCLC from our region, caution is warranted when extrapolating these findings to broader populations. In addition, treatment exposure in our cohort reflects the study period and design, during which the use of contemporary chemo-immunotherapy combinations and targeted therapies was less widespread; therefore, the applicability of these findings to current treatment paradigms may be partially limited. Furthermore, the reduction in patient numbers in mutation subtype–based subgroup analyses inevitably limited statistical power. For the same reason, formal interaction analyses were not performed, as the small numbers within several KRAS subtype categories could have resulted in model overfitting and unstable estimates. This constraint is particularly relevant to comparisons between KRAS G12C and non-G12C subgroups, where numerically observed differences may not have reached statistical significance. Notably, KRAS mutation subtypes did not emerge as independent prognostic factors in survival analyses. This observation suggests that the clinical impact of KRAS subtypes may not be direct, but rather mediated through coexisting clinical characteristics, co-mutation profiles, and the tumor immune microenvironment. The inconsistent results reported in the literature further support the presence of substantial biological heterogeneity among KRAS-mutant tumors. Accordingly, our findings should be considered hypothesis-generating, and prospective, multicenter studies with more comprehensive molecular characterization are required to better define the prognostic and therapeutic relevance of KRAS mutation subtypes.

Overall, our findings highlight that KRAS-mutant NSCLC should not be considered a single biological entity, but rather a heterogeneous group of tumors shaped by distinct mutation subtypes, co-mutation patterns, and immune context. In this real-world cohort, both clinical presentation and molecular background appeared to contribute to variability in outcomes, underscoring the need for a more nuanced, subtype-informed approach to patient stratification. Taken together, these observations support the growing recognition that integrating molecular and clinical features may be essential for improving prognostic assessment and guiding future therapeutic strategies in KRAS-mutant NSCLC.

## 5. Conclusions

In conclusion, our findings support the notion that KRAS-mutant NSCLC represents a biologically heterogeneous disease rather than a single molecular entity. In this real-world cohort, KRAS mutation subtypes, co-mutation patterns, and immune features appeared to be associated with variability in clinical outcomes, although the independent prognostic impact of KRAS subtype was not confirmed in multivariate analysis. These observations underscore the importance of a more integrative, subtype-informed approach to prognostic assessment and highlight the need for prospective, molecularly stratified studies to better define treatment strategies in this patient population.

## Figures and Tables

**Table 1:** Baseline clinical and demographic characteristics of the study population (n = 150).

Variable	n (%) or Median
Age at diagnosis, years, median	62 years (range, 38–83)
**Sex, n (%)**	
Male	126 (84.0)
Female	24 (16.0)
**Smoking history n (%)**	
Current smoker	84 (56.0)
Former smoker	45 (30.0)
Never smoker	21 (14.0)
**ECOG performance status, n (%)**	
0	61 (40.7)
1	79 (52.7)
2	9 (6.0)
3	1 (0.7)
**Disease stage at diagnosis, n (%)**	
Stage I	6 (4.0)
Stage II	5 (3.3)
Stage III	39 (26.0)
Stage IV	100 (66.7)
**First-line treatment (for stage IV disease)**	
Chemotherapy	93.0%
Immunotherapy + chemotherapy	7.0%
**Second-line treatment received**	**79 (52.7)**
**Unable to receive 2nd-line due to death/progression (for stage IV disease)**	**71 (47.3)**
**KRAS mutation subtypes, n (%)**	
G12C	61 (40.7)
G12V	31 (20.7)
G12D	22 (14.7)
G12A	11 (7.3)
G13	11 (7.3)
Other KRAS variants	14 (9.3)
**Co-mutations**	
TP53	15 (10.0)
PIK3CA	18 (12.0)
STK11	8 (5.3)
KEAP1	4 (2.7)
**PD-L1 expression (Tumor Proportion Score), n (%)**	
<1%	70 (46.7)
1–49%	48 (32.0)
≥50%	22 (14.7)
Unknown *	10 (6.7)
**PD-L1 positive (≥1%), n (%)**	**70 (46.7)**
**Metastatic site involvement, n (%)**	
- Brain	62 (41.3)
- Bone	72 (48.0)
- Pleura/contralateral lung	79 (52.7)
- Visceral (liver/adrenal/others)	80 (53.3)
- Distant lymph nodes	66 (44.0)

Abbreviations: ECOG, Eastern Cooperative Oncology Group; S. * Unknown PD-L1 status indicates cases in which PD-L1 testing had not been performed. The indentation should be retained, as it helps distinguish subgroup categories from main variables and improves table readability. The background shading and bold formatting are used to distinguish main variable headings from subgroup categories and improve table readability.

**Table 2:** Univariate Cox regression analysis of overall survival.

Variable	B	*p*	HR (Exp(B))	95% CI (Lower–Upper)
Age	0.038	**0.002**	1.038	1.014–1.063
ECOG performance status	0.639	**<0.001**	1.894	1.394–2.572
Stage at diagnosis	0.610	**0.001**	1.841	1.301–2.604
PD-L1 positivity	−0.399	**0.036**	0.671	0.462–0.975
Sex	−0.237	0.338	0.789	0.486–1.281
KRAS G12C mutation	−0.007	0.971	0.993	0.692–1.425
TP53 mutation	0.308	0.378	1.361	0.686–2.700
Distant lymph node metastasis	−0.268	0.147	0.765	0.533–1.099
Bone metastasis	0.201	0.262	1.223	0.860–1.739
Visceral metastasis	0.023	0.899	1.023	0.717–1.460
Pleural/contralateral lung metastasis	−0.183	0.315	0.833	0.583–1.190
Brain metastasis	−0.037	0.838	0.964	0.677–1.372

Abbreviations: B, regression coefficient; CI, confidence interval; ECOG, Eastern Cooperative Oncology Group; Exp(B), exponentiated regression coefficient; HR, hazard ratio; PD-L1, programmed death-ligand 1. Bold formatting should be retained only for statistically significant *p* values to improve readability.

## Data Availability

The data supporting the findings of this study are available within the article. Further inquiries can be directed to the corresponding author. Correspondence and requests for materials should be addressed to Gökhan Öztürk (E-mail: gokymd@gmail.com).
